# Photobiomodulation Treatment Parameters and Clinical Outcomes Following Periodontal Soft-Tissue Surgery: A Systematic Review

**DOI:** 10.7759/cureus.117156

**Published:** 2026-09-29

**Authors:** Kratee Sharma, Shalabh Mehrotra

**Affiliations:** 1 Department of Periodontics, Teerthanker Mahaveer Dental College and Research Centre, Teerthanker Mahaveer University, Moradabad, IND

**Keywords:** low-level laser therapy, periodontal surgery, photobiomodulation, postoperative pain, wound healing

## Abstract

Photobiomodulation (PBM) has been increasingly investigated as an adjunctive approach to improve postoperative recovery following periodontal surgery. However, substantial variations in treatment parameters and clinical protocols have made it difficult to determine which PBM characteristics are associated with favorable clinical outcomes. This systematic review evaluated the PBM parameters used after periodontal surgery and summarized their reported associations with postoperative clinical outcomes. Randomized controlled clinical studies evaluating PBM after periodontal surgery were identified through a systematic literature search. Studies assessing postoperative pain, wound healing, epithelialization, wound area, graft-related outcomes, and other clinical or biological outcomes were included in this review. Due to substantial heterogeneity in surgical procedures, PBM parameters, application techniques, treatment schedules, and outcome assessments, a quantitative meta-analysis was not performed. Sixteen studies published between 1993 and 2026 were included in this review. Wavelengths ranged from 660 to 970 nm, while power, fluence, application mode, treatment duration, and treatment frequency varied considerably. Overall, PBM has demonstrated potentially favorable effects on wound healing, epithelialization, wound area reduction, and postoperative pain in several studies, although the findings were inconsistent. Some studies have reported no significant improvement in pain or healing despite PBM application. The incomplete reporting of treatment parameters and methodological limitations further restricted the comparison between protocols. Therefore, the available evidence does not establish a specific wavelength, dose, or treatment schedule as optimal. PBM may be a useful adjunct for postoperative periodontal care; however, standardized protocols and adequately powered randomized controlled trials with comprehensive dosimetric reporting are required.

## Introduction and background

Periodontal surgical procedures are routinely performed to manage periodontal defects, facilitate regenerative therapy, and improve soft tissue conditions. Although these procedures can provide predictable clinical benefits, postoperative pain, inflammation, edema, and delayed wound healing can affect patient comfort and recovery [1]. Photobiomodulation (PBM), also known as low-level light or laser therapy, has emerged as a noninvasive adjunctive approach for modulating biological responses following surgical procedures [2]. PBM has been proposed to promote tissue repair, reduce inflammation, and alleviate postoperative pain [2-4]. Clinical studies have reported the beneficial effects of PBM following periodontal surgery, particularly with respect to early wound healing and postoperative pain [3,4].

However, the clinical application of PBM is complicated by the substantial variability in treatment protocols. Studies have used different wavelengths, power outputs, energy densities, exposure times, spot sizes, application modes, treatment frequencies, and number of irradiation sessions [5]. Published periodontal studies have used wavelengths ranging from approximately 588 to 1064 nm, with wide variations in energy, irradiation time, and delivery technique [3]. Such heterogeneity makes direct comparisons between studies difficult and limits the ability to determine which treatment parameters are most consistently associated with favorable clinical outcomes.

Despite the increasing interest in PBM as an adjunct to surgical care, its clinical application remains challenging because treatment outcomes may be influenced by multiple irradiation parameters. Zein et al. [5] highlighted the considerable complexity of PBM and reported the absence of consensus regarding optimal treatment protocols, with substantial variation in wavelength, power, irradiance, energy, fluence, pulse mode, treatment duration, and treatment repetition. They further demonstrated that both insufficient and excessive light doses may result in ineffective responses, thereby emphasizing the importance of appropriate parameter selection. However, this evidence was derived from a broad range of experimental and clinical contexts involving different tissues and biological models; therefore, it cannot be directly extrapolated to periodontal surgical procedures. In periodontal surgery, differences in tissue characteristics, surgical interventions, treatment sites, and clinical outcomes may influence the response to PBM. Consequently, although the previous literature establishes the importance of PBM parameters in determining treatment efficacy, it remains unclear which parameter combinations are most consistently associated with favorable postoperative outcomes, specifically in patients undergoing periodontal surgery. Therefore, a systematic evaluation of the periodontal literature is warranted to characterize the parameters currently used, assess their relationship with postoperative outcomes, and identify inconsistencies and gaps that may guide the development of more standardized PBM protocols.

Accordingly, this systematic review aimed to evaluate the PBM treatment parameters used following periodontal soft-tissue surgery, resulting in secondary-intention wound healing, and to examine their reported associations with postoperative clinical outcomes. Specifically, the review sought to characterize the principal PBM parameters, including wavelength, power output, irradiance, energy, fluence, exposure time, spot size, application mode, treatment frequency, timing of application, and number of treatment sessions; summarize the clinical outcomes reported with different PBM protocols, particularly postoperative wound healing and pain; assess the consistency of reported associations between PBM parameters and clinical outcomes; and identify variations, inconsistencies, and evidence gaps in the current PBM treatment protocols.

## Review

Materials and methods

Review Design and Reporting

This systematic review was conducted in accordance with the Preferred Reporting Items for Systematic Reviews and Meta-Analyses (PRISMA) 2020 statement [6]. This review evaluated the PBM parameters used following periodontal soft-tissue surgery that resulted in secondary-intention wound healing and examined their reported associations with postoperative clinical outcomes. Only randomized controlled trials (RCTs), including parallel-group and split-mouth trials, were considered eligible for inclusion.

Review Question and PICOS Framework

The review question was formulated using the population, intervention, comparator, outcomes, and study design (PICOS) framework [7]. The population comprised human patients who underwent periodontal soft-tissue surgical procedures resulting in secondary intention wound healing. The intervention was postoperative PBM delivered using a low-level laser, diode laser, light-emitting diode (LED), or another clinically applied nonablative light source. Comparators included sham or placebo PBM, no PBM, conventional postoperative care, and alternative PBM protocols. The primary outcome was postoperative soft tissue wound healing, including epithelialization, re-epithelialization, wound closure, and validated healing indices. Secondary outcomes included postoperative pain, analgesic consumption, inflammation, edema, swelling, postoperative morbidity, and patient-reported outcomes. Eligible study designs included randomized controlled clinical trials, including parallel-group and split-mouth RCTs.

Eligibility Criteria

Studies were included if they evaluated human participants undergoing periodontal soft-tissue procedures, such as gingivectomy, gingivoplasty, surgical gingival depigmentation, free gingival grafting, or connective tissue grafting, in which the donor or surgical wound was allowed to heal by secondary intention. PBM had to be administered postoperatively or as an adjunct to surgery, and the study had to report at least one clinically relevant postoperative outcome with sufficient information on at least one treatment parameter. Randomized pilot trials were eligible when true random allocation was used.

Studies were excluded if they involved animals, in vitro models, non-periodontal surgical procedures, implant surgery, extraction sockets, or wound healing primarily by closure. Conventional periodontal flap or regenerative procedures were excluded if they did not produce a predominantly secondary intention soft-tissue wound. Studies in which lasers or light were used for cutting, ablation, de-epithelialization, coagulation, or other surgical purposes rather than PBM were also excluded. Non-randomized clinical studies, observational studies, cohort studies, case-control studies, case series, case reports, conference abstracts without sufficient data, protocols, editorials, letters, and review articles were excluded from the study. Studies with insufficient information to determine the PBM protocol or relevant clinical outcomes were excluded. When multiple publications reported the same trial population, they were assessed for overlap and treated as a single study.

Information Sources

Electronic searches were conducted in MEDLINE via PubMed, Scopus, Web of Science Core Collection, Embase, Cochrane Central Register of Controlled Trials (CENTRAL), and Cumulative Index of Nursing and Allied Health Literature (CINAHL). Google Scholar was searched, and the reference lists of the included studies and relevant systematic reviews were screened manually. Forward citation searches were also performed for key eligible studies. The databases were searched from inception to August 1, 2026, without restrictions on the publication year.

Search Strategy

A comprehensive literature search was conducted from database inception to August 1, 2026, in the databases specified above. The search strategy was developed using combinations of controlled vocabulary and free-text terms related to photobiomodulation, low-level laser therapy, periodontal soft-tissue surgery, and postoperative clinical outcomes. Boolean operators “AND” and “OR” were used to combine the search concepts. The search strategy was adapted to the syntax and indexing requirements of each database. No restrictions were applied based on publication year. The search strategy was designed to maximize sensitivity and identify studies evaluating photobiomodulation following periodontal soft-tissue surgical procedures. The search strategy was developed based on the concepts of PBM and periodontal soft-tissue surgery. As the terminology for PBM has evolved over time, both contemporary and historical terms were included. PBM-related terms included “photobiomodulation,” “photobiomodulation therapy,” “low-level laser therapy,” “low-level light therapy,” “low-intensity laser therapy,” “LLLT,” “LILT,” “soft laser therapy,” “therapeutic laser,” “laser biostimulation,” “laser biomodulation,” “light therapy,” “LED therapy,” and “light-emitting diode.” Periodontal terms included “periodontal surgery,” “gingivectomy,” “gingivoplasty,” “gingival surgery,” “gingival depigmentation,” “free gingival graft,” “gingival graft,” “connective tissue graft,” “palatal wound,” “palatal donor site,” and “graft donor site.”

The primary search was intentionally not restricted by PBM parameter terminology because wavelength, power, fluence, irradiance, energy, and exposure characteristics were frequently reported only in the full texts. Parameter-related terms, including “wavelength,” “fluence,” “irradiance,” “energy density,” “power density,” “dose,” “energy,” “power,” “spot size,” “exposure time,” “continuous,” and “pulsed,” were used during supplementary searching and full-text assessment. The search strategy was adapted to the indexing system and syntax of each database.

Study Selection

The retrieved records were imported into reference-management software, and duplicate records were removed. Two reviewers (KS and SM) independently screened the titles and abstracts according to the predefined eligibility criteria. Potentially relevant records were retrieved in full text and independently assessed by both reviewers. Particular attention was given to confirmation of randomization, the type of periodontal surgical procedure, secondary-intention wound healing, postoperative administration of PBM, and availability of treatment-parameter information. Disagreements were resolved through discussion and consensus. Reasons for exclusion at the full-text stage were documented, and the study-selection process is presented in a PRISMA 2020 flow diagram (Figure 1).

**Figure 1:**
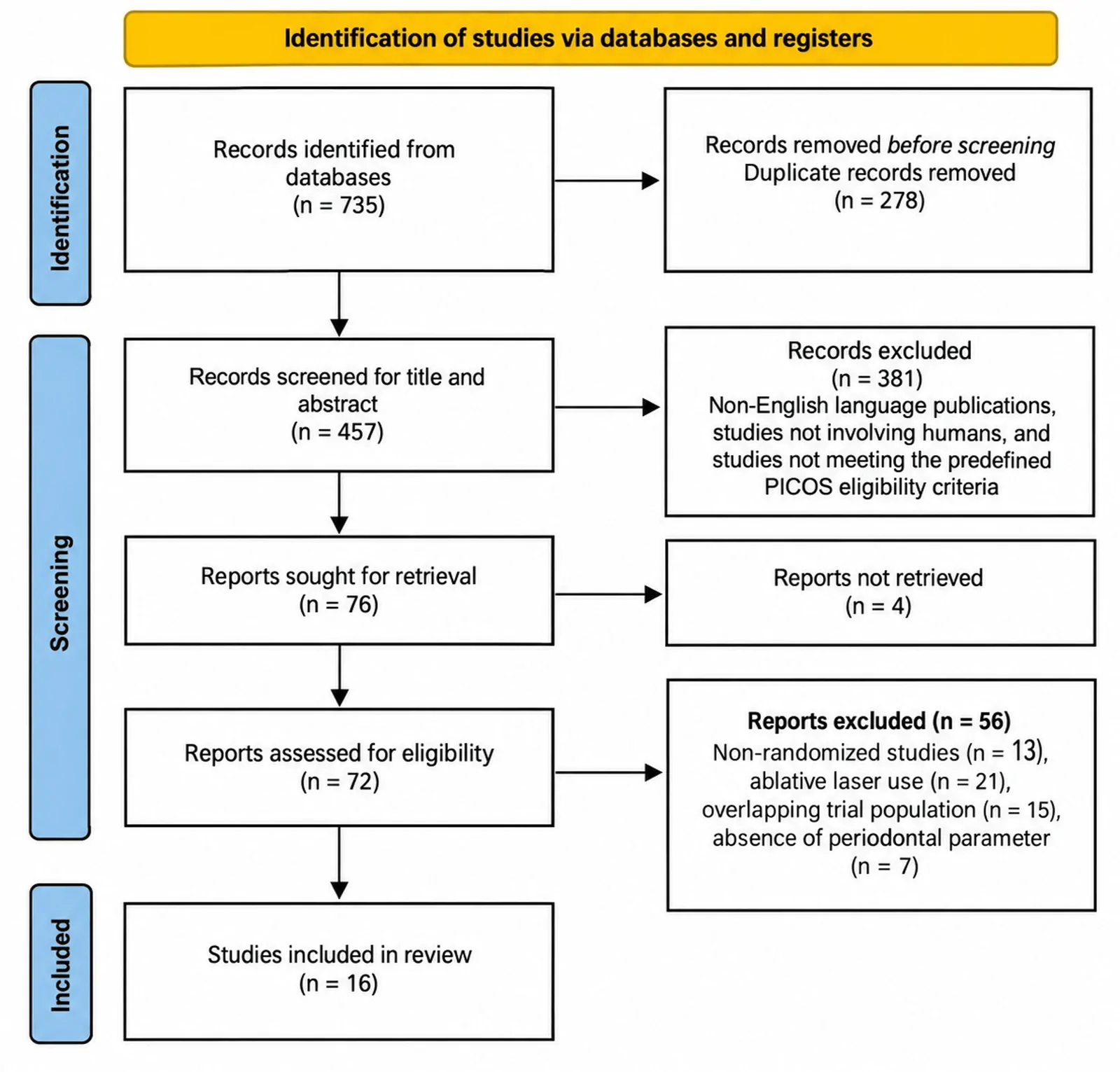
Preferred Reporting Items for Systematic Reviews and Meta-Analyses (PRISMA) 2020 flow diagram showing the identification, screening, eligibility assessment, and inclusion of studies in the systematic review. PICOS: population, intervention, comparator, outcomes, and study design; n: number of records, reports, or studies, as applicable.

Data Extraction

The extracted study characteristics included the author, publication year, country, study design, sample size, number of treated sites, clinical setting, and follow-up duration. The participant characteristics included age, sex, periodontal condition, and relevant clinical characteristics. Surgical information included the type of procedure, surgical technique, anatomical site, treatment area, graft or donor site characteristics, suturing or dressing procedures, and standard postoperative care.

Detailed PBM information was extracted for each treatment arm and site. The recorded parameters included the device and manufacturer, light source, wavelength, emission mode, power output, irradiance, energy per point or session, fluence, spot size, exposure time, number of irradiation points, treatment area, contact or non-contact application, tissue-to-device distance, number and interval of sessions, timing of the first application, and total treatment duration. Parameters were recorded using the original units reported by the respective authors. Where adequate information permitted the calculation of missing dosimetric variables, the derived values were calculated separately and identified as reviewer-derived data.

Outcome Assessment

The primary outcome was postoperative soft tissue wound healing. Data were extracted for clinical healing indices, wound area reduction, epithelialization, re-epithelialization, percentage healing, and other validated measures of wound repair. Secondary outcomes included postoperative pain, analgesic consumption, inflammation, edema, swelling, postoperative morbidity, tissue-related outcomes, and patient-reported outcome measures. The assessment instrument, measurement method, postoperative time point, and effect direction were recorded for each outcome.

Risk of Bias Assessment

The risk of bias was assessed independently by two reviewers using the Cochrane Risk of Bias 2 (RoB 2) tool [8]. The assessment covered bias arising from the randomization process, deviations from the intended interventions, missing outcome data, measurement of outcomes, and selection of reported results. For split-mouth trials, the assessment additionally considered the appropriateness of treatment allocation, potential contamination between treatment sites, independence of outcome measurements, and whether the paired nature of the data was appropriately considered in the analyses. Each domain was classified as having a low risk of bias, some concerns, or a high risk of bias according to the RoB 2 framework. Disagreements were resolved through discussion and consultation.

Data Synthesis

A meta-analysis was not performed because the included RCTs demonstrated substantial clinical and methodological heterogeneity. The studies varied in terms of surgical procedure, wound characteristics, PBM device, wavelength, power, irradiance, energy, fluence, spot size, exposure duration, treatment frequency, number and timing of sessions, comparator intervention, outcome definition, assessment method, and follow-up duration. These differences prevented meaningful quantitative pooling and made the calculation of a common treatment effect inappropriate.

Assessment of the Certainty of Evidence

The certainty of evidence for the principal outcomes was assessed using the Grading of Recommendations Assessment, Development, and Evaluation (GRADE) framework, where sufficient evidence was available [9]. The assessment considered the risk of bias, inconsistency, indirectness, imprecision, and other relevant factors. As no meta-analysis was performed, certainty judgments were based on the consistency and methodological quality of the available randomized evidence and the overall coherence of the parameter-outcome results.

Results

Study Characteristics

Sixteen studies were included in the systematic review, published between 1993 and 2026. The studies evaluated PBM following different periodontal surgical procedures, including mucogingival surgery, gingivoplasty, gingivectomy, gingival depigmentation, free gingival graft (FGG) procedures, connective tissue graft donor-site wounds, and periodontal flap surgery [10-25]. The study designs included randomized controlled, split-mouth, crossover, sham-controlled, placebo-controlled, and three-arm clinical trials. The sample sizes ranged from 10 to 40 participants, with most studies involving relatively small populations. Masse et al. [10] evaluated soft laser treatment following mucogingival periodontal surgery in a double-blind crossover randomized clinical trial involving 28 paired procedures. No significant differences were observed in pain, gingival inflammation, or healing between the laser-treated and placebo sites. Damante et al. [11] similarly found no significant acceleration of clinical or histomorphometric healing after gingivoplasty. Almeida et al. [12] evaluated PBM at FGG recipient sites and reported no significant improvement in healing or analgesic outcomes compared with sham irradiation (Table 1).

**Table 1:** Characteristics of the included studies. RCT, randomized controlled trial; PBM, photobiomodulation; LLLT, low-level laser therapy; FGG, free gingival graft; CTG, connective tissue graft; DE-FGF, de-epithelialized free gingival graft; VAS, visual analog scale; OHIP-14, Oral Health Impact Profile-14; OHRQoL, oral health-related quality of life; PPD, probing pocket depth; CAL, clinical attachment level; NS, non-significant; PBMT, photobiomodulation therapy; AlGaAs, aluminium gallium arsenide; As-Ga, arsenide-gallium; He-Ne, helium-neon; InGaAsP, indium gallium arsenide phosphide.

Author, publication year	Country	Study design and sample	Surgical procedure/site	PBM intervention	Comparator	Outcomes and follow-up	Principal finding
Masse et al., 1993 [10]	Canada	Double-blind crossover randomized clinical trial; n = 28 paired procedures	Mucogingival periodontal surgery	As-Ga and He-Ne soft laser	Contralateral placebo	Pain; gingival inflammation; healing (days 7 and 14)	No significant benefit
Damante et al., 2004 [11]	Brazil	Split-mouth clinical/histomorphometric study; n = 16 histomorphometry; companion clinical report	Gingivoplasty	670-nm AlGaAs LLLT	Contralateral non-irradiated site	Histomorphometry; clinical healing	No significant acceleration of healing
Almeida et al., 2009 [12]	Brazil	Split-mouth sham-controlled double-blind RCT; n = 10	FGG recipient site	Dual-wavelength AlGaAs LLLT	Sham laser	Photographic healing; pain/analgesic use	No significant healing or analgesic benefit
Dias et al., 2015 [13]	Brazil	Parallel randomized sham-controlled trial; n = 32	CTG palatal donor site	660-nm AlGaAs LLLT	Sham LLLT	Wound area; tissue thickness; discomfort	Smaller wound area at selected follow-ups
Chawla et al., 2016 [14]	India	Double-blind placebo-controlled split-mouth study; 12 patients, 30 sites	Surgical gingival depigmentation	810-nm defocused diode LLLT	Contralateral control	Wound area/epithelialization; VAS	Benefit mainly at day 3
Ustaoglu et al., 2017 [15]	Turkey	Parallel randomized controlled study; n = 40	FGG palatal donor site	940-nm AlGaAs LLLT	Control	Epithelialization; healing; color; bleeding; pain; thickness	Favorable healing-related findings
Heidari et al., 2017 [16]	Iran	Split-mouth placebo-controlled triple-blind RCT; n = 12	FGG palatal donor/recipient sites	660-nm diode LLLT	Laser-off placebo	Epithelialization; pain	Epithelialization favored LLLT; pain not improved
Isler et al., 2018 [17]	Turkey	Parallel 3-arm RCT; n = 36	FGG palatal donor site	970±15-nm diode LLLT	Spontaneous-healing control; ozone arm	Epithelialization; wound area; VAS	No clear wound-area benefit; pain benefit at day 7
Kohale et al., 2018 [18]	India	Randomized controlled split-mouth study; n = 40	Scalpel gingivectomy	940-nm InGaAsP diode LLLT	Laser-off control	Epithelialization; healing index; VAS	Healing and pain favored LLLT
Lingamaneni et al., 2019 [19]	India	Randomized double-blind split-mouth pilot; n = 10	Gingivectomy/gingivoplasty	810-nm diode LLLT	Sham laser	Healing index; wound surface to day 14	Healing better by day 14
Yildiz and Gunpinar, 2019 [20]	Turkey	Parallel placebo-controlled triple-masked RCT; n = 30	FGG recipient site	810-nm AlGaAs LLLT	Laser-off placebo	Graft shrinkage; epithelialization; VAS; analgesics	Shrinkage and pain favored PBM; epithelialization NS
Uslu and Akgül, 2020 [21]	Turkey	Parallel 3-arm RCT; n = 36	Gingivectomy/gingivoplasty	810-nm AlGaAs PBM	No-PBM control; ozone arm	VAS; OHIP-14	Pain/OHRQoL favored PBM
Madi and Mahmoud, 2020 [22]	Saudi Arabia	Randomized controlled parallel study; n = 20	Scalpel gingivectomy	660-nm diode LLLT	No LLLT	Healing index; VAS	Healing and pain favored LLLT
Morshedzadeh et al., 2022 [23]	Iran	Randomized triple-blind split-mouth RCT; 16 recruited, 14 analyzed	FGG palatal donor site	940-nm AlGaAs PBM	Sham irradiation	Wound area; epithelialization; pain; bleeding; color	Wound area favored PBM; pain NS
Paz et al., 2025 [24]	Brazil	Parallel RCT; n = 30	DE-FGF palatal donor site	Dual-wavelength PBMT + fibrin sponge	Fibrin sponge alone	Wound area; epithelialization; VAS; analgesics	Day-10 epithelialization favored PBMT; other outcomes NS
Sharma and Mehrotra, 2026 [25]	India	Prospective parallel-group pilot RCT; n = 20	Periodontal flap surgery	LLLT + cyanoacrylate adhesive	Conventional silk sutures	Pain; wound-healing index; analgesics; PPD; CAL	Pain/analgesic use and early healing favored combined intervention

Photobiomodulation Parameters

Marked variations were observed in the PBM parameters used across the 16 studies. Wavelengths ranged from 660 to 970 nm, with 660, 810, and 940 nm being the most frequently reported wavelengths. Some studies have used dual-wavelength protocols. The output power varied from 15 mW to 3 W, and the reported fluence also differed substantially between studies. Continuous-wave delivery has been commonly reported, although the mode of application, contact versus non-contact technique, irradiation points, spot size, treatment duration, and treatment frequency varied considerably.

Damante et al. [11] used a 670-nm aluminium gallium arsenide (AlGaAs) laser at 15 mW and 4 J/cm² per point, delivered by punctual contact irradiation at three points over four sessions at 48-hour intervals. Almeida et al. [12] used dual wavelengths of 660 and 780 nm at 40 mW and 10 J/cm². Dias et al. [13] used 660-nm irradiation at 30 mW, whereas Chawla et al. [14] used an 810-nm defocused diode laser at 1 W. Ustaoglu et al. [15] and Kohale et al. [18] used 940-nm protocols, although their treatment parameters and delivery approaches differed. Several studies did not provide complete PBM dosimetry data. Power and fluence were unavailable in the extracted information for Masse et al. [10], while incomplete parameter reporting was also evident for Paz et al. [24] and Sharma & Mehrotra [25]. Heidari et al. [16] reported inconsistent fluence values, and some parameters for Isler et al. [17] were marked as requiring confirmation. Accordingly, unavailable parameters were recorded as not reported rather than being inferred (Table 2).

**Table 2:** Photobiomodulation parameters and reported clinical outcomes. NR, not reported or not available in the extracted source; CW, continuous wave; PBM, photobiomodulation; LLLT, low-level laser therapy; PBMT, photobiomodulation therapy; OHRQoL, oral health-related quality of life; VAS, visual analog scale; NS, not statistically significant; As-Ga, arsenide-gallium; He-Ne, helium-neon. Missing parameters were recorded as NR and were not inferred.

Author (reference no.)	Wavelength (nm)	Power	Fluence	Mode/application	Sessions	Principal finding
Masse et al. [10]	As-Ga; He-Ne	NR	NR	Soft laser; exact application details NR	NR	Pain, inflammation and healing: no significant benefit
Damante et al. [11]	670	15 mW	4 J/cm²/point	CW; punctual/contact; 3 points	4; every 48 h	No acceleration of healing
Almeida et al. [12]	660 + 780	40 mW	10 J/cm²	Punctual; 20 s; geometry NR	2; 0 and 48 h	Healing/pain NS
Dias et al. [13]	660	30 mW	3 J/cm²/point; 15 J/cm²/session	CW; punctual/contact; 5 points	Registry: 8; every 48 h	Wound area smaller at selected follow-ups
Chawla et al. [14]	810	1 W	NR	CW; defocused; noncontact	Daily through day 7	Wound-area benefit at day 3
Ustaoglu et al. [15]	940	3 W	8.6 J/cm²/session	CW reported; noncontact; 2.8-cm² probe	4; days 0, 3, 5, 7	Healing-related outcomes favored LLLT
Heidari et al. [16]	660	200 mW	4 J/cm² stated; 32 J/cm² in discussion	CW; noncontact; 0.5-cm spot	5; days 0, 1, 2, 4, 7	Epithelialization favored PBM; pain NS
Isler et al. [17]	970 ± 15	2 W	35 J/cm²	CW*; 320-µm fiber; 5 points	Days 0, 1, 3, 7	Healing uncertain; pain better on day 7
Kohale et al. [18]	940	100 mW	4 J/cm²/tooth	CW; noncontact 1-2 cm; circular motion	3; days 1, 3, 7	Healing and pain favored LLLT
Lingamaneni et al. [19]	810	100 mW	NR	CW; noncontact; 5 min	4 applications	Healing better by day 14
Yildiz and Gunpinar [20]	810	100 mW	6 J/cm² stated	CW; noncontact/perpendicular; 0.5-cm spot	5; days 0, 1, 3, 7, 14	Shrinkage/pain favored PBM; epithelialization NS
Uslu and Akgül [21]	810	300 mW	4 J/cm²	CW; noncontact; 1 min/application	3; days 0, 3, 7	Pain/OHRQoL favored PBM
Madi and Mahmoud [22]	660	50 mW	4 J/cm²	CW; noncontact; 3-mm beam; 1-cm distance	3; days 0, 3, 5	Healing/VAS favored LLLT
Morshedzadeh et al. [23]	940	0.21 W	5 J/cm²	CW; noncontact 2-3 mm; 2.8-cm² probe	6; every 48 h	Wound area favored PBM; pain NS
Paz et al. [24]	Dual wavelength; exact pair NR	NR	NR	PBMT; geometry/dose NR	5; days 0, 3, 7, 10, 15	Day-10 epithelialization favored PBMT
Sharma and Mehrotra [25]	NR	NR	NR	LLLT + cyanoacrylate; laser details NR	NR	Pain/early healing favored combined intervention

Postoperative Pain

Postoperative pain was one of the most frequently assessed outcomes, although the results were inconsistent. Masse et al. [10] reported no significant pain reduction with soft laser treatment. Almeida et al. [12] similarly found no significant analgesic benefits. Heidari et al. [16] reported improved epithelialization but no significant improvement in postoperative pain, while Morshedzadeh et al. [23] found a favorable effect on wound area without a significant difference in pain. In contrast, Isler et al. [17] reported lower pain at day 7, whereas Kohale et al. [18] found that both healing and pain outcomes favored low-level laser therapy (LLLT) following gingivectomy. Yildiz and Gunpinar [20] reported reduced pain at the FGG recipient site, and Uslu and Akgül [21] reported favorable effects on pain and oral health-related QoL. Madi and Mahmoud [22] reported improvements in pain following gingivectomy. Sharma and Mehrotra [25] reported lower postoperative pain and analgesic consumption following periodontal flap surgery; however, their intervention combined LLLT with cyanoacrylate adhesive (Table 1).

Wound Healing and Epithelialization

The findings for wound healing were generally more favorable than those for pain, although considerable variations were observed. Damante et al. [11] found no significant acceleration of healing after gingivoplasty. Chawla et al. [14] reported a wound-area benefit at day 3 following gingival depigmentation. Kohale et al. [18] reported improved healing and epithelialization following gingivectomy, whereas Lingamaneni et al. [19] observed better healing by day 14 following gingivectomy/gingivoplasty. Among palatal donor-site studies, Dias et al. [13] reported smaller wound areas at selected follow-up points. Ustaoglu et al. [15] reported favorable healing-related outcomes, and Heidari et al. [16] found improved epithelialization despite no significant pain reduction. Isler et al. [17] did not demonstrate a clear wound area benefit, although pain was lower on day 7. Morshedzadeh et al. [23] reported a favorable effect of PBM on wound area, whereas Paz et al. [24] observed improved epithelialization at day 10, with other assessed outcomes showing no significant differences (Table 1).

Other Clinical and Biological Outcomes

Yildiz and Gunpinar [20] reported reduced graft shrinkage at the FGG recipient site, although epithelialization did not differ significantly between the groups. Uslu and Akgül [21] reported improvements in oral health-related quality of life, in addition to pain. Damante et al. [11] included histomorphometric evaluation but did not demonstrate accelerated histological healing (Table 1).

Risk of Bias

The risk of bias assessment indicated that most of the included studies had some concerns. Although several trials used sham or placebo irradiation and reported blinding, concerns remained regarding randomization procedures, allocation methods, incomplete reporting, outcome assessment, and selective reporting. Masse et al. [10], Damante et al. [11], Almeida et al. [12], Dias et al. [13], and several subsequent studies were judged to have some overall concerns. Kohale et al. [18] and Morshedzadeh et al. [23] had relatively favorable assessments in several domains, whereas Paz et al. [24] had concerns across multiple domains. Sharma and Mehrotra [25] were judged to have low risk in the randomization and missing-data domains, but some concerns in other domains, particularly because there was no sham LLLT and the intervention included cyanoacrylate as a co-intervention (Figure 2). No included study was judged to have a high overall risk of bias. However, several studies had some concerns in one or more individual RoB 2 domains, with Paz et al. [24] showing some concerns across all assessed domains.

**Figure 2:**
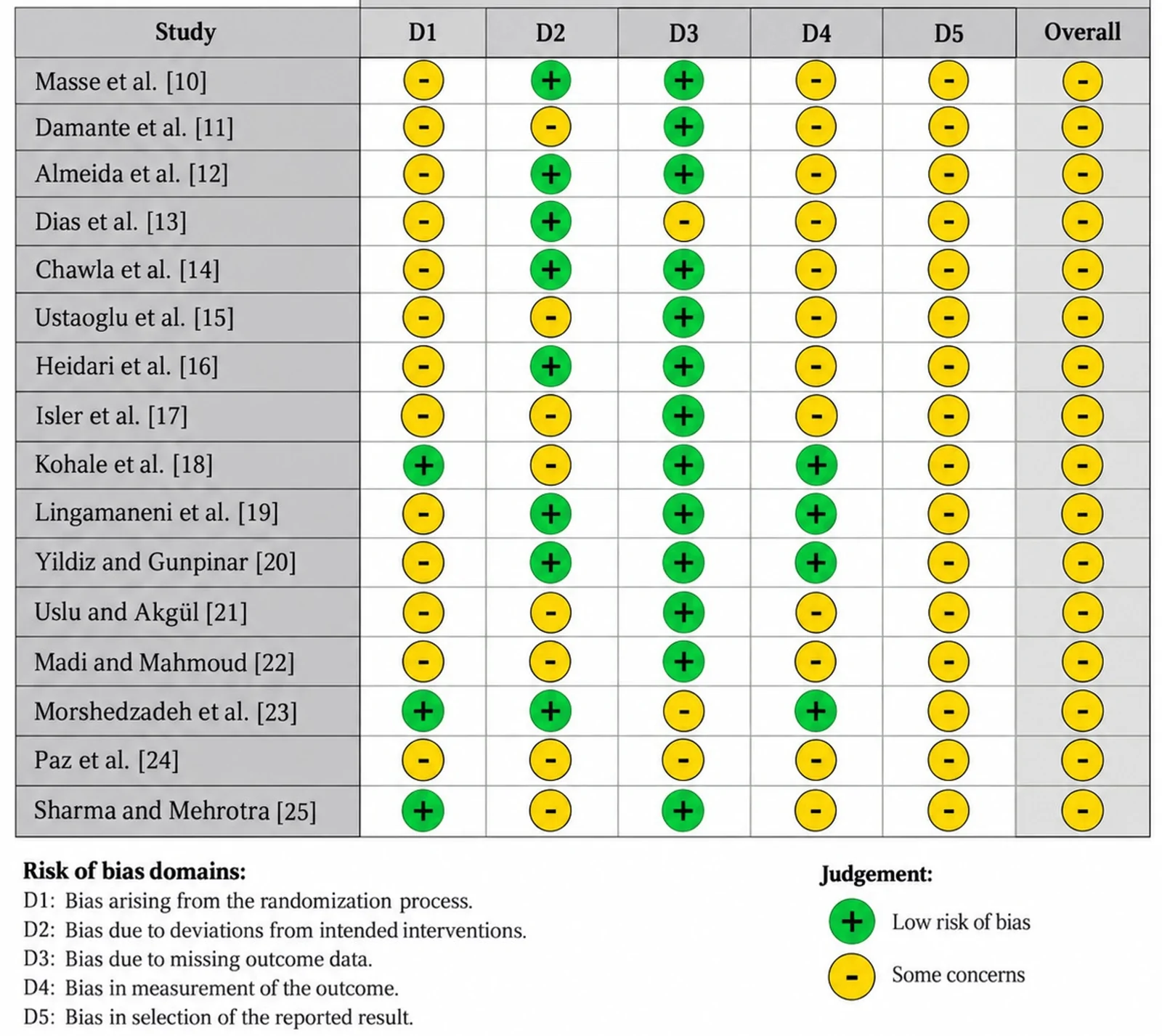
Risk of bias summary for the included randomized controlled studies using the revised Cochrane Risk of Bias 2 (RoB 2) tool. Green circles indicate low risk of bias, whereas yellow circles indicate some concerns. The “Overall” column represents the overall risk-of-bias judgment for each study. No included study was judged to have a high risk of bias. The risk-of-bias judgments were based on the five RoB 2 domains specified above [8].

Certainty of Evidence

The certainty of the evidence was judged to be very low across the reported outcome domains. The principal factors limiting certainty were methodological concerns, variations in PBM protocols, differences in surgical procedures and wound models, incomplete reporting of treatment parameters, and small sample sizes. As the present review did not perform a meta-analysis, the certainty assessment was interpreted qualitatively rather than on the basis of pooled effect estimates or statistical heterogeneity (Table 3).

**Table 3:** Summary of the certainty of evidence using the GRADE approach. GRADE, Grading of Recommendations Assessment, Development, and Evaluation; FGG, free gingival graft; OHRQoL, oral health-related quality of life. Certainty was downgraded where methodological limitations, inconsistent findings, indirectness, or small sample sizes were considered important.

Author (reference no.)	Main outcome domain	Risk of bias	Inconsistency	Indirectness	Imprecision	Certainty
Masse et al. [10]	Pain; inflammation; healing	Some concerns	Serious	Serious	Serious	Very low
Damante et al. [11]	Clinical/histologic healing	Some concerns	Serious	Serious	Serious	Very low
Almeida et al. [12]	Healing; pain	Some concerns	Serious	Serious	Serious	Very low
Dias et al. [13]	Palatal wound area; discomfort	Some concerns	Serious	Serious	Serious	Very low
Chawla et al. [14]	Depigmentation wound healing; pain	Some concerns	Serious	Serious	Serious	Very low
Ustaoglu et al. [15]	Palatal epithelialization; healing	Some concerns	Serious	Serious	Serious	Very low
Heidari et al. [16]	Epithelialization; pain	Some concerns	Serious	Serious	Serious	Very low
Isler et al. [17]	Palatal epithelialization; wound area; pain	Some concerns	Serious	Serious	Serious	Very low
Kohale et al. [18]	Gingivectomy healing; pain	Some concerns	Serious	Serious	Serious	Very low
Lingamaneni et al. [19]	Gingivectomy/gingivoplasty healing	Some concerns	Serious	Serious	Serious	Very low
Yildiz and Gunpinar [20]	FGG recipient-site shrinkage; pain	Some concerns	Serious	Serious	Serious	Very low
Uslu and Akgül [21]	Pain; OHRQoL	Some concerns	Serious	Serious	Serious	Very low
Madi and Mahmoud [22]	Gingivectomy healing; pain	Some concerns	Serious	Serious	Serious	Very low
Morshedzadeh et al. [23]	Palatal wound area; epithelialization; pain	Some concerns	Serious	Serious	Serious	Very low
Paz et al. [24]	Palatal epithelialization; wound area; pain	Some concerns	Serious	Serious	Serious	Very low
Sharma and Mehrotra [25]	Flap-surgery healing; pain	Some concerns	Serious	Very serious	Very serious	Very low

Overall Findings

Overall, the 16 included studies demonstrated inconsistent but potentially favorable effects of PBM on selected postoperative outcomes. Benefits were reported more consistently for wound healing, epithelialization, and wound area reduction than for postoperative pain. However, several studies have reported no significant benefits, and no consistent relationship has been established between a particular wavelength, power, fluence, application technique, or treatment frequency and superior clinical outcomes. The substantial variation in PBM protocols and surgical contexts, together with incomplete reporting of treatment parameters, limits the ability to identify a standardized protocol associated with consistently favorable outcomes.

Discussion

Principal Findings

The present systematic review demonstrated that PBM may have beneficial effects on selected postoperative outcomes following periodontal and related soft-tissue surgical procedures, particularly wound healing, epithelialization, wound area reduction, and postoperative pain. However, the findings were not uniform across studies, and no single PBM protocol was identified as consistently superior. The principal reason for this inconsistency appears to be the substantial variation in both the clinical surgical models and the parameters used to deliver PBM. This finding is important because PBM should not be regarded simply as an intervention that is either effective or ineffective; rather, its biological response depends on the interaction between the wavelength, irradiance, fluence, exposure time, treatment frequency, tissue characteristics, and timing of application. The complexity of these interactions has been emphasized by Zein et al. [5], who demonstrated considerable variability in effective and ineffective PBM doses across experimental models.

Hadis et al. [26] highlighted that incomplete or inaccurate reporting of PBM parameters, particularly power, beam area, irradiance, radiant energy, and treatment characteristics, can substantially compromise reproducibility and comparison across studies. This is particularly relevant to the present review, in which several included studies had incomplete dosimetric reporting. Jenkins and Carroll [27] emphasized that accurate reporting of wavelength, power, irradiation time, beam area, pulse parameters, treatment location, number of treatments, and treatment intervals is essential for reproducibility of LLLT studies. They also recommended reporting dose-related measures, such as energy and energy density, together with the application technique and beam characteristics, where possible.

Influence of Surgical Procedure and Wound Characteristics

The clinical context appears to be an important determinant of treatment response. The included studies investigated gingivoplasty, gingivectomy, gingival depigmentation, FGG procedures, connective tissue graft donor sites, and periodontal flap surgeries [10-25]. These procedures generate wounds that differ substantially in size, depth, vascularity, tissue manipulation, healing mechanisms, and exposure to the oral environment. Consequently, the same PBM dose cannot be expected to produce equivalent biological effects across different surgical sites.

The most consistent favorable findings were observed in studies involving palatal donor site wounds. Dias et al. [13], Ustaoglu et al. [15], Heidari et al. [16], Morshedzadeh et al. [23], and Paz et al. [24] reported improvements in at least one measure of wound healing or epithelialization. These wounds heal predominantly by secondary intention, providing a clinically relevant model for evaluating epithelialization and tissue repair. In contrast, Almeida et al. [12] found no significant improvement in healing at FGG recipient sites, suggesting that PBM may have different effects depending on whether the tissue is an open donor wound or graft recipient bed.

Chawla et al. [14] reported an early benefit at day 3, whereas Lingamaneni et al. [19] detected a significant difference only at day 14. This temporal difference may reflect the biological stages of wound healing in the body. PBM has been proposed to modulate inflammatory signaling and cellular activity during the early stages of repair, while subsequent epithelial migration, fibroblast proliferation, extracellular matrix deposition, and remodeling may determine the later clinical appearance [2,5,27]. The findings from gingivectomy and gingivoplasty also support the importance of the healing phase, in which the outcome is measured [28]. Therefore, an intervention may produce an early biological effect that is not necessarily maintained throughout the healing period.

Influence of Wavelength

Considerable variation was observed in wavelength, ranging from approximately 660 to 970 nm, with 660, 810, and 940 nm being the most frequently represented. Damante et al. [11] used 670 nm, Almeida et al. [12] used 660/780 nm, Dias et al. [13] used 660 nm, Chawla et al. [14] used 810 nm, Ustaoglu et al. [15] used 940 nm, and subsequent studies employed 660-, 810-, 940-, and approximately 970-nm protocols [16-25]. This variation may influence the tissue penetration and interaction with cellular chromophores. However, the present evidence does not support the conclusion that a particular wavelength is universally optimal for all plant species. Zein et al. [5] emphasized that PBM efficacy cannot be interpreted according to wavelength alone because multiple optical parameters interact to determine the biological response. Similarly, Nowak-Terpiłowska et al. [29] demonstrated that the biological response of human gingival fibroblasts varied according to the combined effects of wavelength, power density, and energy density, with different parameter combinations resulting in different cellular responses. Thus, the favorable findings associated with 810 or 940 nm in some studies should not be interpreted as evidence of superiority over 660 nm wavelength. Differences in power, fluence, treatment area, irradiation distance, and treatment frequency occurred simultaneously across the included clinical studies and prevented the isolation of wavelength as an independent determinant.

Influence of Dose and Treatment Parameters

Dose variation was an even more prominent source of this heterogeneity. The included studies used powers ranging from approximately 15 mW to 3 W, with different fluences and treatment schedules [10-25]. For example, Damante et al. [11] used 15 mW and 4 J/cm² per point but did not demonstrate accelerated healing, whereas other protocols using substantially different power and irradiation arrangements reported favorable outcomes. This should not be interpreted as evidence that a higher power produces better healing. Power, fluence, irradiance, spot size, exposure time, and treatment area are interdependent, and increasing one parameter without considering the others can substantially alter the biological dose.

The biphasic dose-response concept is particularly relevant to the findings of this review. Huang et al. [30] described that PBM may demonstrate a biphasic response, in which lower light doses can produce greater stimulatory and tissue-repair effects than higher doses. They further emphasized that variations in wavelength, fluence, power density, pulse structure, and treatment timing may contribute to both positive and negative treatment outcomes. Zein et al. [5] similarly highlighted the wide variability in effective and ineffective PBM parameters and the possibility of reduced responses with excessive dosing. These observations provide a plausible explanation for why apparently similar PBM protocols may produce different clinical outcomes and why increasing the dose does not necessarily result in greater clinical benefits. In the present review, the inability to calculate or directly compare complete dosimetric profiles for all the included studies represented an important limitation.

Treatment Frequency and Timing

The treatment frequency also varied substantially, from single or limited applications to repeated irradiation over several postoperative days [10-25]. This may influence outcomes because the biological response of a surgical wound changes rapidly from inflammation to proliferation and remodeling [31]. Repeated PBM may theoretically influence more than one stage of healing; however, excessive or unnecessarily prolonged irradiation may not provide additional benefits. Consequently, the number and timing of sessions should be considered together with the dose delivered at each session, rather than being treated as independent protocol characteristics [2].

Clinical Outcomes and Possible Mechanisms

The relatively favorable findings for wound healing are biologically plausible. PBM has been proposed to influence mitochondrial activity and cellular signaling, potentially supporting fibroblast proliferation, angiogenesis, collagen production, and modulation of inflammatory mediators [2]. These findings are also supported by Basso et al. [32], who reported that LLLT enhanced oral fibroblast migration and proliferation and stimulated the expression of wound-healing-related genes following exposure to inflammatory cytokines. These findings suggest that PBM may promote periodontal wound repair by modulating fibroblast activity and growth factor signaling.

Pain outcomes were inconsistent. Some studies reported reduced postoperative pain, including Yildiz and Gunpinar [20], Uslu and Akgül [21], Madi and Mahmoud [22], and Sharma and Mehrotra [25], whereas Almeida et al. [12], Heidari et al. [16], and Morshedzadeh et al. [23] did not demonstrate consistent analgesic effects. This discrepancy may be related to differences in surgical trauma, analgesic use, timing of pain assessment, and the subjective nature of patient-reported pain. Earlier systematic reviews have also evaluated postoperative pain and wound healing following PBM as adjunctive periodontal treatment, but the presence of substantial heterogeneity supports the need for more standardized protocols [3,4].

Clinical Implications

From a clinical perspective, these findings suggest that PBM may be considered a potential adjunct rather than a universally established postoperative treatment. The strongest practical rationale appears to be for selected soft-tissue wounds, in which delayed epithelialization, postoperative discomfort, or wound area reduction is clinically important. However, clinicians should avoid selecting protocols solely based on wavelength or device availability. Treatment parameters should be reported and interpreted as a complete dosimetric profile, including the wavelength, output power, irradiance, fluence, spot size, exposure duration, irradiation mode, treatment points, distance from the tissue, timing of the first application, and number of sessions. The present evidence does not justify the recommendation of a standardized dose for all periodontal surgical procedures.

Limitations and Future Recommendations

This review has several limitations. The included studies were clinically heterogeneous with respect to surgical procedure, wound type, PBM wavelength, power, fluence, application technique, treatment frequency, comparator, and follow-up period. Several studies incompletely reported important PBM parameters, while some contained inconsistencies in reported dosimetry or treatment sessions. Small sample sizes and methodological concerns further reduced confidence in the findings. In addition, some studies incorporated co-interventions, such as fibrin sponges or cyanoacrylate adhesives, making it difficult to attribute the observed effect exclusively to PBM [24,25]. The present review did not perform a meta-analysis because the clinical and methodological heterogeneity was considered too substantial for meaningful quantitative pooling. One included study was authored by members of the review team, which may have limited the independence of study selection and methodological appraisal and should be considered when interpreting the findings [25].

Future RCTs should therefore use standardized and completely reported PBM protocols, preferably with sham irradiation, adequate allocation concealment and blinding, predefined clinically relevant outcomes, and a sufficiently long follow-up. Importantly, investigators should report the wavelength, power, irradiance, fluence, spot size, exposure time, treatment area, application mode, treatment distance, timing, and number of sessions in accordance with reproducible PBM reporting principles [5]. Future research should directly compare different parameter combinations within the same surgical model to determine whether specific dose ranges are associated with superior wound healing or pain outcomes. Larger multicenter trials and prospectively registered protocols are required before a clinically standardized PBM protocol can be recommended.

## Conclusions

PBM may provide beneficial effects on selected postoperative outcomes following periodontal surgery, particularly wound healing, epithelialization, and pain. However, substantial variation in wavelength, power, fluence, application technique, treatment frequency, and surgical site prevents the identification of an optimal protocol. Incomplete parameter reporting and methodological limitations further reduce the certainty. Therefore, PBM may be considered a promising adjunct; however, standardized, adequately powered RCTs with comprehensive dosimetric reporting are required to establish reproducible and clinically applicable treatment protocols.
